# Computational and Spectroscopic Studies of Carbon Disulfide

**DOI:** 10.3390/molecules25081901

**Published:** 2020-04-20

**Authors:** Indri B. Adilina, Fauzan Aulia, Muhammad A. Fitriady, Ferensa Oemry, Robert R. Widjaya, Stewart F. Parker

**Affiliations:** 1 Research Center for Chemistry, Indonesian Institute of Sciences, Kawasan Puspiptek Serpong, Tangerang Selatan, Banten 15314, Indonesia; indri.badria.adilina@lipi.go.id (I.B.A.); fauzan.aulia@lipi.go.id (F.A.); muhammad.arifuddin.fitriady@lipi.go.id (M.A.F.); robert.ronal.widjaya@lipi.go.id (R.R.W.); 2 Research Center for Physics, Indonesian Institute of Sciences, Kawasan Puspiptek Serpong, Tangerang Selatan, Banten 15314, Indonesia; ferensa.oemry@lipi.go.id; 3 ISIS Facility, STFC Rutherford Appleton Laboratory, Chilton, Didcot, Oxon OX11 0QX, UK

**Keywords:** carbon disulfide, inelastic neutron scattering, Raman spectroscopy, infrared spectroscopy, density functional theory

## Abstract

The vibrational spectroscopy of CS_2_ has been investigated many times in all three phases. However, there is still some ambiguity about the location of two of the modes in the solid state. The aim of this work was to locate all of the modes by inelastic neutron scattering (INS) spectroscopy, (which has no selection rules), and to use periodic density functional theory to provide a complete and unambiguous assignment of all the modes in the solid state. A comparison of the observed and calculated INS spectra shows generally good agreement. All four of the *ν*_2_ bending mode components are calculated to fall within 14 cm^−1^. Inspection of the spectrum shows that there are no bands close to the intense feature at 390 cm^−1^ (assigned to *ν*_2_); this very strongly indicates that the *A_u_* mode is within the envelope of the 390 cm^−1^ band. Based on a simulation of the band shape of the 390 cm^−1^ feature, the most likely position of the optically forbidden component of the *ν*_2_ bending mode is 393 ± 2 cm^−1^. The calculations show that the optically inactive *A_u_* translational mode is strongly dispersed, so it does not result in a single feature in the INS spectrum.

## 1. Introduction

Carbon disulfide, CS_2_, changed from being a laboratory curiosity in the mid-1800s when it was first used industrially for the vulcanization of rubber. Since then, it has had many commercial uses. The current major uses are for the production of viscose rayon and cellophane and as a solvent in adhesives and cleaners [1]. 

The vibrational spectroscopy of CS_2_ has been investigated many times in all three phases [2,3,4,5,6,7,8,9,10,11,12]. However, there is still some ambiguity about the location of two of the modes in the solid state. The aim of this work was to locate all of the modes by inelastic neutron scattering (INS) spectroscopy, which has no selection rules [13], and to use periodic density functional theory to provide a complete and unambiguous assignment of all the modes in the solid state. 

## 2. Results

**Figure 1:**
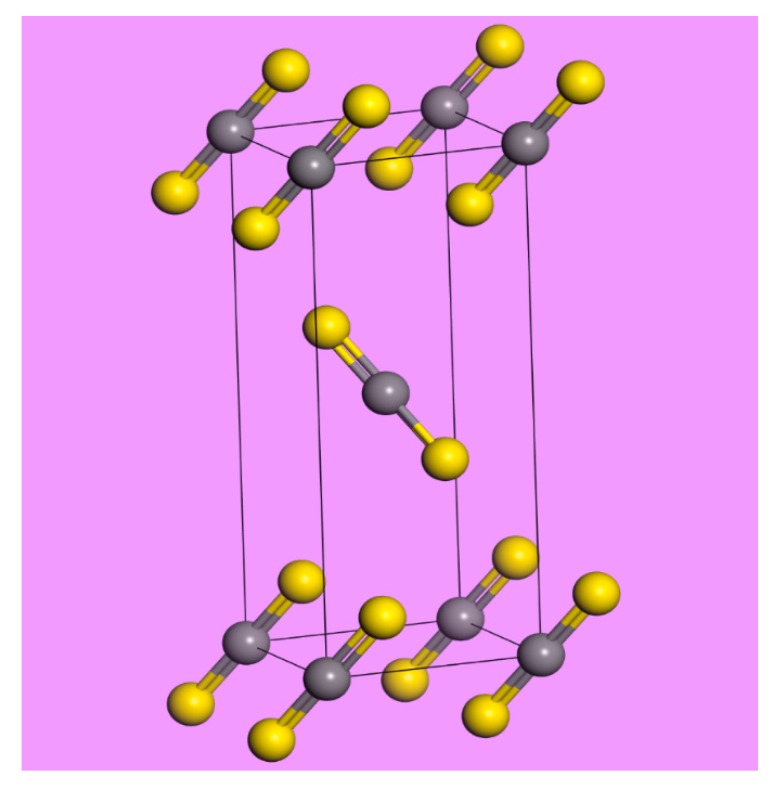
The primitive unit cell of carbon disulfide. Grey = carbon, yellow = sulfur.

**Figure 2:**
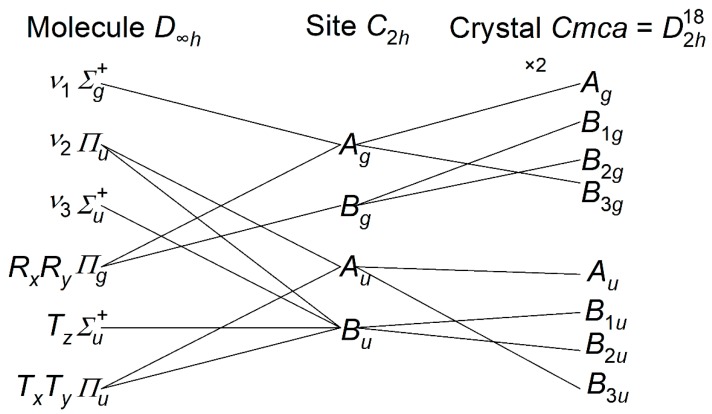
Correlation table for carbon disulfide in the solid state. *R* = libration, *T* = translation.

In the solid state, the crystal is orthorhombic (*Cmca*) with two molecules in the primitive unit cell, both on sites of *C*_2*h*_ symmetry [14,15]; see Figure 1. Figure 2 shows the correlation diagram for CS_2_ and that, at the Γ-point in the Brillouin zone (where the infrared and Raman active modes occur), the modes comprise:

{*A_g_* + *B*_3*g*_} *ν*_1_ symmetric stretch, {*A_u_* + *B*_1*u*_ + *B*_2*u*_ + *B*_3*u*_} *ν*_2_ bend,
{*B*_1*u*_ + *B*_2u_} *ν*_3_ asymmetric stretch, {*A_g_* + *B*_1*g*_ + *B*_2*g*_ + *B*_3*g*_} libration,
{*B*_1*g*_ + *B*_2*g*_} translation along *z*, {*A_u_* + *B*_1*u*_ + *B*_2*u*_ + *B*_3*u*_} translation along *x,y*

In addition, there are three acoustic translational modes that have zero energy at the Γ-point, but non-zero elsewhere in the Brillouin zone. These are not observable by infrared or Raman spectroscopy; however, INS can measure these [10,11]. All *g* modes are Raman active; *B*_1*u*_, *B*_2*u*_, *B*_3*u*_ are infrared active, and *A_u_* is inactive in both the infrared and the Raman. Note that all modes are allowed in the INS spectrum.

**Figure 3:**
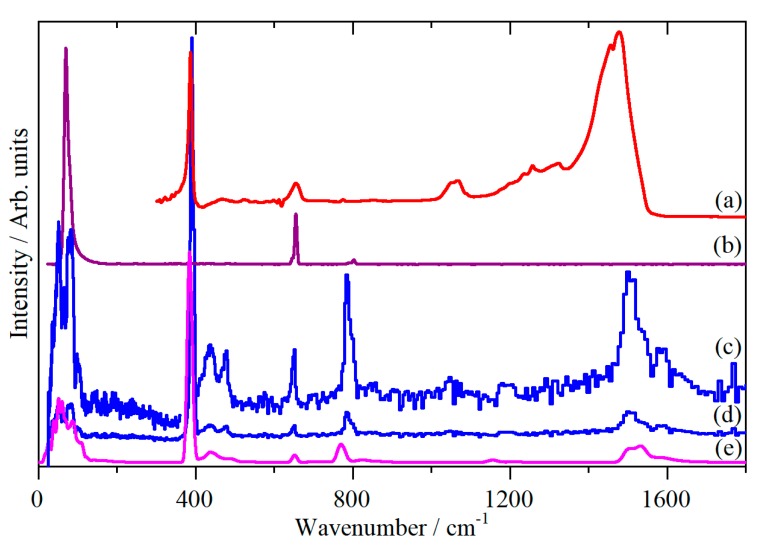
Vibrational spectra of solid CS_2_. (a) infrared at 208 K, (b) FT-Raman at ~ 77 K, (c) INS at 20 K, ×5 ordinate expansion, (d) INS at 20 K, and (e) INS spectrum generated from a periodic density functional theory (DFT) calculation of CS_2_.

Figure 3 shows the infrared, Fourier transform Raman (FT-Raman), and INS spectra. The infrared and Raman spectra are in agreement with those previously published [7], and the INS spectrum has not been seen previously. The internal fundamental modes are clearly seen at 1494 cm^−1^ (*ν*_3_ asymmetric stretch), 655 cm^−1^ (*ν*_1_ symmetric stretch), and 390 cm^−1^ (*ν*_2_ bend). The external (lattice) modes all occur below 150 cm^−1^. The remaining features are: *ν*_3_ + lattice (1586 cm^−1^), *ν*_1_ + *ν*_2_ (1066/1052 cm^−1^), 2 *ν*_2_, (788 cm^−1^), and *ν*_2_ + lattice (476/440 cm^−1^). Table 1 lists the fundamental modes.

There have been several attempts at detailed assignments of the spectra by lattice dynamics; however, these have all been based on empirical force field models [7,10,11,16,17]. Figure 3e shows the INS spectrum generated from a periodic density functional theory (DFT) calculation of the primitive unit cell, which includes the effect of vibrational dispersion (the variation of transition energy with wavevector). It can be seen that there is generally good agreement. Table 1 also lists the transition energies of the modes (and their symmetry) at the Γ-point in the Brillouin zone (where the infrared and Raman active modes are observed).

The calculations show that the inactive *A_u_* component of the *ν*_2_ bend quartet is almost coincident with one of the allowed modes. The observed modes are ~10 cm^−1^ higher in energy, but the separation between the highest and lowest member, for both the observed and calculated transition energies, is almost the same: 11.4 and 14.0 cm^−1^, respectively. The full width at half maximum of the very strong INS band at 390 cm^−1^ is 11.9 cm^−1^, and there are no other features close by. As all four components of *ν*_2_ will have similar INS intensity (because the motion is the same in each case, only the phasing differs, and this is confirmed by the ACLIMAX [18] calculation of the individual intensities), this very strongly indicates that the *A_u_* mode is within the envelope of the 390 cm^−1^ band.

**Figure 4:**
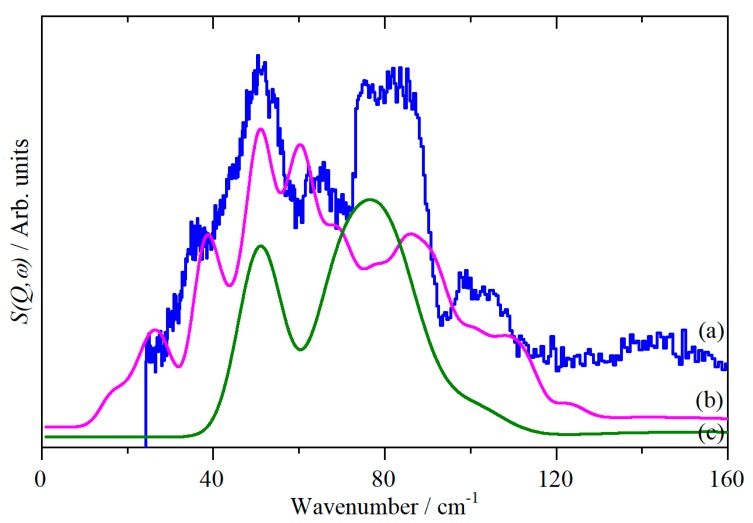
INS spectra of solid CS_2_ in the lattice mode region. (a) Observed, (b) generated from a periodic-DFT calculation for the complete Brillouin zone, and (c) as (b) but for the Γ-point only.

**Figure 5:**
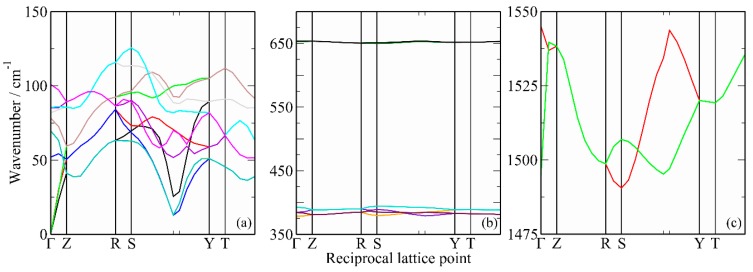
Calculated dispersion curves of solid CS_2_. (**a**) In the lattice mode region, (**b**) *ν*_2_ and *ν*_1_, and (**c**) *ν*_3_.

Figure 4 presents a detailed comparison of the observed and calculated INS spectra in the low energy region. It is evident that there is a considerable difference between the full dispersion calculation, 4b, and the Γ-point only calculation, 4c, demonstrating that there is significant dispersion present, as seen for the acoustic modes by inelastic coherent neutron scattering from a single crystal [11]. The calculated dispersion curves are shown in Figure 5. It can be seen that all of the modes in the region below 150 cm^−1^ exhibit significant dispersion, which makes the association of specific features in the INS spectrum, in this region, with a particular mode meaningless. The “missing” translational mode is calculated to be at 52 cm^−1^ at the Γ-point, but disperses over the range 20–80 cm^−1^; thus, it makes a contribution to the structured peak at 51 cm^−1^, but this also includes significant contributions from acoustic translational modes. This is apparent from the additional peaks at 26 and 39 cm^−1^ in the full dispersion calculation, 4b, that are absent in the Γ-point only calculation, 4c, as the acoustic modes have zero energy at the Γ-point, Figure 5a.

The comparison of Figure 4b and 4c also shows that the features at 99 and 104 cm^−1^, that have not been previously reported, are a consequence of the dispersion in the librational modes away from the Γ-point, Figure 5a.

## 3. Discussion

The aim at the outset of this project was to locate the two infrared and Raman inactive factor group components of the translations and the *ν*_2_ bending mode. The expectation was that, because all the modes are allowed in the INS, this should enable their location, as has been done for many other systems, e.g., C_60_ [19] and M(CO)_6_ (M = Cr, Mo, W) [20]. This has proven to be more complicated than anticipated. The width of an INS peak is determined by the instrumental resolution and the inherent peak width, which depends on the vibrational dispersion. All four of the *ν*_2_ bending mode components fall within 12 cm^−1^ and have a few wavenumbers dispersion, which accounts for the measured width (the resolution at this energy transfer is ~5 cm^−1^ [13]). Based on a simulation of the band shape of the 390 cm^−1^ feature, the most likely position of the optically forbidden component of the *ν*_2_ bending mode is 393 ± 2 cm^−1^. 

The translational mode is much more problematic. The calculations, Figure 4a, show that this is strongly dispersed, so it does not result in a single feature in the INS spectrum. The calculated value of 52 cm^−1^ at the Γ-point is slightly higher than the 37 cm^−1^ measured close to the melting point of CS_2_ [11], but the latter is likely to be severely affected by anharmonicity.

## 4. Materials and Methods 

Carbon disulfide (99%) was purchased from Sigma-Aldrich (Gillingham, Dorset, UK) and used as received.

INS spectra were recorded using the TOSCA [21] spectrometer at the ISIS Pulsed Neutron and Muon Facility (Chilton, Oxfordshire, UK) [22]. On TOSCA, the resolution is ~1.25% of the energy transfer across the entire energy range. Infrared spectra (4 cm^−1^ resolution, 64 scans) were recorded between 105 K and 298 K with a Bruker Vertex 70 Fourier transform infrared spectrometer using a Specac single reflection variable temperature attenuated total internal reflection accessory. The FT-Raman spectrum was recorded at room temperature and 77 K from the sample inside a quartz cuvette with a Bruker MultiRam spectrometer using 1064 nm excitation (500 mW laser power and 1024 scans at 4 cm^−1^ resolution).

Dispersion corrected periodic density functional theory (DFT-D) calculations were carried out using the plane wave pseudopotential method, as implemented in the CASTEP code (version 17.21) [23,24]. Exchange and correlation were approximated using the PBE [25] functional with the Tkatchenko-Scheffler (TS) dispersion correction scheme [26] within the generalized gradient approximation (GGA). The plane-wave cut-off energy was 750 eV. Brillouin zone sampling of electronic states was performed on a 12 × 12 × 4 Monkhorst-Pack grid (84 k-points). The equilibrium structure, an essential prerequisite for lattice dynamics calculations, was obtained by BFGS geometry optimization after which the residual forces were converged to ±0.00087 eV Å^−1^. Phonon frequencies were obtained by diagonalization of the dynamical matrix, computed using density-functional perturbation theory [27], to compute the dielectric response and the Born effective charges, and, from these, the mode oscillator strength tensor and infrared absorptivity were calculated. In addition to the calculation of transition energies and intensities at zero wavevector, phonon dispersion was also calculated along high symmetry directions throughout the Brillouin zone. For this purpose, dynamical matrices were computed on a regular grid of wavevectors throughout the Brillouin zone, and Fourier interpolation was used to extend the computed grid to the desired fine set of points along the high-symmetry paths [28]. The atomic displacements in each mode, that are part of the CASTEP output, enable visualization of the modes to aid assignments and are also all that is required to generate the INS spectrum using the program ACLIMAX (version 6.0.0 LE) [18]. It is emphasised that, for the calculated spectra and dispersion curves shown, the transition energies have not been scaled.

## 5. Conclusions

This paper highlights the crucial interplay of theory and experiment for vibrational spectroscopy. Without the calculations, finding the location of the *A_u_* component of the *ν*_2_ bend quartet would be simply guesswork, as none of the three forms of vibrational spectroscopy enable observation of the mode, but it must contribute to the INS spectrum. Similarly for the *A_u_* translational mode, the calculations show this to be highly dispersed, so a distinct feature cannot be assigned to it. The comparison of the theory and the experiment for the INS spectra illustrates the power of the combination, but also demonstrates that DFT still has some way to go before it can predict intermolecular modes with the same reliability that it does for intramolecular modes.

## Figures and Tables

**Table 1:** Observed and calculated (at the Γ-point) transition energies and intensities of the fundamental modes of CS_2_.

DFT				INS ^1^	Infrared	Raman	Description
/ cm^−1^	Symmetry	Infrared / Debye^2^ Å^−2^ amu^−1^	Raman / Å^4^ amu^−1^	/ cm^−1^	/ cm^−1^	/ cm^−1^	
0.0	*B*_2*u*_	0.000	0.0				Acoustic
0.0	*B*_1*u*_	0.000	0.0				Acoustic
0.0	*B*_3*u*_	0.000	0.0				Acoustic
52.0	*A_u_*	0.000	0.0	51			Translation
69.7	*B*_1*u*_	0.007	0.0		66.5 [9]		Translation
78.1	*B*_2*u*_	0.007	0.0		68.2 [9]		Translation
81.9	*A_g_*	0.000	231.0			75 [7]	Libration
85.3	*B*_1*g*_	0.000	21.9			79 [7]	Libration
85.6	*B*_3*g*_	0.000	23.2			79 [7]	Libration
100.9	*B*_2*g*_	0.000	109.8			85 [7]	Libration
378.3	*B*_3*u*_	0.202	0.0		388.7 [8]		*ν*_2_ bend
384.0	*B*_2*u*_	0.197	0.0		393.4 [8]		*ν*_2_ bend
384.5	*A_u_*	0.000	0.0				*ν*_2_ bend
392.3	*B*_1*u*_	0.225	0.0	390 vs	400.1 [8]		*ν*_2_ bend
653.1	*A_g_*	0.000	913.1	651 w		655 s	*ν*_1_ symmetric stretch
653.9	*B*_3*g*_	0.000	68.2			646 m	*ν*_1_ symmetric stretch
1494.6	*B*_1*u*_	42.570	0.0	1507 w	1479 vs		*ν*_3_ asymmetric stretch
1536.3	*B*_2*u*_	10.132	0.0	1540 w	1530 sh		*ν*_3_ asymmetric stretch

^1^ s = strong, m = medium, w = weak, v = very, sh = shoulder.
